# Quantitating Fluorescence Intensity From Fluorophore: Assignment of MESF Values

**DOI:** 10.6028/jres.110.010

**Published:** 2005-04-01

**Authors:** A. K. Gaigalas, Lili Wang, Abe Schwartz, Gerald E. Marti, Robert F. Vogt

**Affiliations:** National Institute of Standards and Technology, Gaithersburg, MD 20899-8312; Center for Quantitative Cytometry PO Box 19344, San Juan, PR 00919; Center for Biologics Evaluation and Research, FDA Bethesda, MD 20892; Division of Laboratory Sciences, CDC Atlanta, GA 30341

**Keywords:** cytometry, FITC, fluorescein, fluorescence yield, MESF, microspheres

## Abstract

A procedure is presented to convert the comparison of measured fluorescence signals into a comparison of fluorescence yields (FY). The fluorescence yield, which is a property of a solution or a suspension, is defined as the product of the fluorophore concentration and the molecular quantum yield. The paper revises the measurement model which relates the measured fluorescence signal to the FY. The equality of FY of two solutions provides an equivalence between the concentrations of fluorophore in the two solutions. The equivalence is the basis for quantitation in terms of molecules of equivalent soluble fluorophore (*MESF*). The quantitation procedure starts with the measurement of fluorescence signals from a serial dilution of fluorescein solutions to obtain a calibration of a fluorometer. The fluorometer is used to measure the fluorescence signal of a suspension of microspheres with immobilized fluorescein isothiocyanate (FITC). The calibration is used to obtain the concentration of soluble fluorophores which gives the same fluorescence signal as the microsphere suspension. The number concentration of microspheres is measured and the equality of fluorescence yields is used to obtain the number of soluble fluorescein molecules equivalent to a single microsphere.

## 1. Introduction

RM 8640 consists of six plastic bottles each containing a 2 mL suspension of polymethyl methacrylate (PMMA) microspheres with a specified amount of immobilized fluorescein isothiocyanate (FITC). The microspheres are intended for calibrating the fluorescence response of flow cytometers [1]. This paper describes the procedures used for assigning values of *MESF* (molecules of equivalent soluble fluorophore) to the microspheres with immobilized FITC. There are three major measurements in this procedure. First, the concentration of microspheres is measured using a Multisizer 3 (Coulter Corporation, Miami FL) particle counter^1^. Second, a fluorometer is calibrated using SRM 1932, a fluorescein solution. Third, the fluorescence signal is measured for each of the microsphere suspensions. Finally, the data from the three measurements are used to calculate the *MESF* values of the microspheres.

In practice, we made serial dilutions of SRM 1932 and calibrated the fluorometer response as a function of fluorescein concentration. Since the concentration of fluorescein varies from 10^−12^ mol/L to 10^−9^ mol/L, it was necessary to pay special attention to contamination, linearity, photodegradation, and background subtraction. The measurements of the concentration of the microsphere suspension constitutes the operational definition of particle concentration. There are at present no particle number standards to validate the concentration measurement.

A cytometer was used to measure the fluorescence signals associated with the five microsphere populations each with a different amount of immobilized FITC. A valid assignment of *MESF* values should yield a linear relation between the measured fluorescence signal in the cytometer and the assigned *MESF* values. A linearization procedure was used to impose a linear relation between the cytometer response of the five microsphere populations and their *MESF* values.

## 2. Revised Measurement Model and the Assignment of *MESF*

It was pointed out [2] that the quantum yield as defined in Eqs. (A3) and (A14) in Ref. [3] is the ratio of fluorescent radiant flux to absorbed radiant flux. Since the radiant flux is the product of the number flux and average spectral energy, the ratio of radiant fluxes is not the same as the ratio of number fluxes. In the following we introduce a modification to the measurement model which allows a consistent use of quantum yield, a molecular property defined in terms of number flux.

In the previous paper [3] we expressed the fluorescence spectral radiance, *L*_f_(*λ*_m_,*λ*_x_), as

$$\begin{array}{l} L_{f}\left(\lambda_{m},\lambda_{x}\right)=\frac{S_{f}\left(\lambda_{m}\right)}{S^{\prime }\left(\lambda_{m}\right)}L^{\prime }\left(\lambda_{m}\right)\\ =2.3FNly\left(\lambda_{m},\lambda_{x}\right)\varepsilon \left(\lambda_{x}\right)\Phi_{i}\left(\lambda_{x}\right)\Delta \lambda_{x} \end{array}$$ (1)

*λ*_m_ and *λ*_x_ are the emission and excitation wavelengths, *S*_f_, *S*′, are measured signals, *L*′ is the spectral radiance of a reference source, *F*, *l*, *Φ*_i_(*λ*_x_), and ∆*λ*_x_ are instrument characteristics, and *N*, *y*, and *ε* are sample properties. The quantity *y*(*λ*_m_, *λ*_x_) relates the absorbed radiant flux at wavelength *λ*_x_ to the fluorescent radiant flux at wavelength *λ*_m_. In other words, the radiant flux from fluorescence is a fraction *y* of the absorbed radiant flux. The radiant flux can be converted to a photon number flux by dividing the radiant flux by the energy of a single photon. Thus the quantity *y*(*λ*_m_, *λ*_x_) can be converted into a relation between fluorescence photon number flux and absorbed photon number flux by multiplying it by the ratio of the respective wavelengths.

$$y^{\prime }\left(\lambda_{m},\lambda_{x}\right)=\frac{\lambda_{m}}{\lambda_{x}}y\left(\lambda_{m},\lambda_{x}\right).$$ (2)

The quantity *y*′(*λ*_m_, *λ*_x_) is conveniently separated into a quantum yield *φ* and a normalized relative photon emission function *s*′(*λ*_m_, *λ*_x_) (1/nm), where

$$\int s^{\prime }\left(\lambda_{m},\lambda_{x}\right)d\lambda_{m}=1.$$ (3)

Thus Eq. (1) can be rewritten as

$$\frac{S_{f}\left(\lambda_{m}\right)}{S^{\prime }\left(\lambda_{m}\right)}\frac{\lambda_{m}}{\lambda_{x}}L^{\prime }\left(\lambda_{m}\right)=2.3FNl\phi s^{\prime }\left(\lambda_{m},\lambda_{x}\right)\varepsilon \left(\lambda_{x}\right)\Phi_{i}\left(\lambda_{x}\right)\Delta \lambda_{x}.$$ (4)

Integrating over all emission wavelengths gives

$$\int S_{f}\left(\lambda_{m}\right)\left[\frac{\lambda_{m}}{\lambda_{x}}\frac{L^{\prime }\left(\lambda_{m}\right)}{S^{\prime }\left(\lambda_{m}\right)}\right]d\lambda_{m}=\Omega N\phi \varepsilon \left(\lambda_{x}\right)I_{0}.$$ (5)

The change in the measurement model amounts to a modification of the spectral correction function given by the term in brackets on the left side of Eq. (5). Eq. (5) supplants a similar equation in Ref. [3]. The left side of Eq. (5) is found experimentally by performing the specified operation on the measured fluorescence emission spectrum. The reference source used to calibrate the detector wavelength response was unpolarized. In order to minimize the effects due to polarization of the fluorescence emission, the incident laser beam polarization was confined to the plane defined by the incident and detected light beams. We made no estimate of possible artifacts due to polarization differences.

Suppose that the operation in Eq. (5) is performed on the emission spectrum from a reference solution with known concentration of fluorophore and the emission spectrum from a suspension of microspheres with immobilized FITC. Furthermore, the number concentration of microspheres is known. In the case that the two numbers on the left of Eq. (5) are equal, the corresponding solution and suspension properties on the right side of Eq. (5) are also equal.

$$ΩN_{\text{sol}}\phi_{\text{sol}}\varepsilon_{\text{sol}}\left(\lambda_{x}\right)I_{0}=ΩN_{\text{sus}}\phi_{\text{sus}}\varepsilon_{\text{sus}}\left(\lambda_{x}\right)I_{0}.$$ (6)

The subscripts “sol” and “sus” in Eq. (6) refer to solution and suspension, respectively. We assume that the experimental conditions used for the measurements on solution and suspension are the same and that the solution and suspension have equal indexes of refraction. In that case, *I*_0_ and Ω are the same on both sides of Eq. (6) and can be factored out.

$$N_{\text{sol}}\phi_{\text{sol}}\varepsilon_{\text{sol}}\left(\lambda_{x}\right)=N_{\text{sus}}\phi_{\text{sus}}\varepsilon_{\text{sus}}\left(\lambda_{x}\right).$$ (7)

It is known from measurements that there are shifts in the wavelength of maximum absorption. It is likely that the absolute value of the extinction coefficient is also different. The extinction coefficient of fluorophore immobilized on a microsphere has not been measured due to predominance of scattering. However, we make the major assumption that the molar extinction coefficient is the same for fluorophore in solution and immobilized on the microsphere. Thus, Eq. (7) reduces to an equality of fluorescence yields.

$$N_{\text{sol}}\varphi_{\text{sol}}=N_{\text{sus}}\phi_{\text{sus}}.$$ (8)

Based on Eq. (8)*N*_sol_ is equivalent to *N*_sus_. The calculation used to assign molecules of equivalent soluble fluorophore (*MESF*) values to the microspheres is given by

$$MESF=\frac{N_{A}}{1000}\frac{C_{\text{eq}}}{N_{\text{spheres}}}$$ (9)

where *N*_sus_ = *N*_sphere_ (mL^−1^) is the number concentration of fluorescein labeled microspheres and *C*_eq_ is the molar concentration of soluble fluorescein which gives the same value for the left side of Eq. (5) as the suspension of microspheres. Avogadro’s constant, *N*_A_ (6.022 × 10^23^), in Eq. (9) is a conversion factor between molar and number concentrations. The equivalent concentration of soluble fluorescein is determined using the fluorescein calibration curve

$$C_{\text{eq}}=10^{-\text{intercept}}{\left(FS\times P_{\text{adj}}\right)}^{\text{slope}}$$ (10)

where “intercept” and “slope” are the linear fit parameters describing the relationship between the logarithm of the observed fluorescence signal and the logarithm of the concentration of fluorescein. *FS* is the fluorescence signal of the microsphere suspension evaluated according to the left side of Eq. (5). The value *FS* has to be adjusted for possible differences in illumination intensity between the calibration measurements and the microsphere measurements. The ratio of the average of the power readings taken during the calibration and microsphere measurements is set equal to the adjustment factor, *P*_adj_, which multiplies *FS* in Eq. (10). The power readings varied by less than 1 % during the calibration or the microsphere measurements. However, since the two measurements were taken on different days, the difference in average power could be as high as several percent. Eq. (9) and Eq. (10) constitute the operational definition of the *MESF* assignment.

In the following we describe the procedure used for obtaining the five factors needed in Eq. (9) and Eq. (10). As an example, the value of *MESF* is obtained for one specific suspension of microspheres.

## 3. Calibration of the Fluorometer

**Fig. 1:**
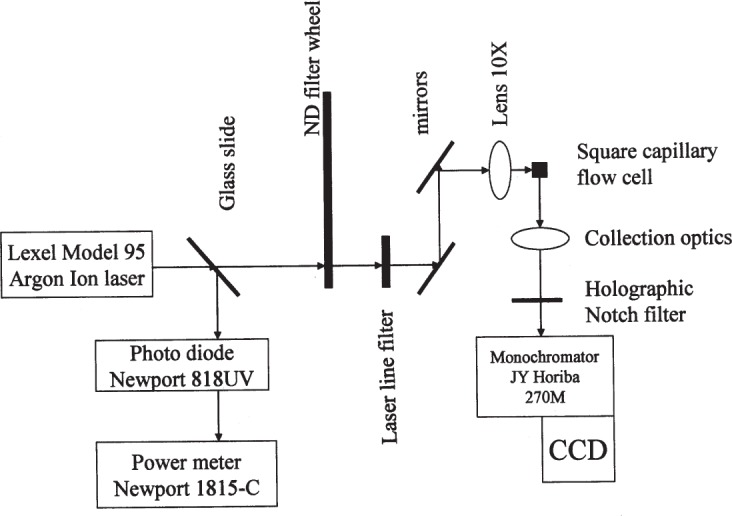
A schematic diagram of the fluorometer used for the assignment of *MESF* values to microspheres, with immobilized fluorescein isothiocyanate, in suspension. The instrument is a modified Raman spectrometer. A square capillary flow cell with 100 µm inside dimension contained the flowing sample. Not shown is a peristaltic pump which pumped the sample from a 1.5 mL vial.

Figure 1 shows a schematic of the fluorometer used in the *MESF* assignments. A water-cooled argon ion laser (Lexel model 95) was the source of 488 nm light. A glass slide reflected a portion of the output beam and directed it towards a photo diode (Newport 818 UV) whose output was processed by a power meter (Newport 1815-C). The accuracy of the power reading was about 2 % (product specifications). The output of the power meter was monitored as an index of relative illumination power which was used to determine the factor *P*_adj_ in Eq. (10). The laser beam, transmitted through the glass slide, passed a neutral density (ND) filter wheel which contained ND filters of nominal optical density (OD) values 0, 1, 2, and 3. The beam passed through a laser line filter to eliminate plasma lines from the laser and then was directed via two mirrors to the entrance aperture of a 10× microscope objective. The two reflections changed the vertical polarization into horizontal. The objective lens focused the laser beam on a square capillary flow cell (WWP100375 from Polymicro Technologies) with a inner dimension of 100 µm. The portion of the capillary that was illuminated by the laser was stripped of the polyimide coating which normally surrounds the glass capillary. The capillary flow cell was mounted on a rotary stage which in turn was mounted on a *X*-*Y* translation stage. The rotary stage was used to position the capillary so that the incident beam was perpendicular to one of the sides. The *X*-*Y* stage was used to position the capillary at the position of the monochromator entrance slit image.

The Model 270M monochromator, made by JY Horiba, was equipped with a CCD-3000 Detector System. The CCD chip used in the measurements was back illuminated with a minimum quantum efficiency of 65 % at 550 nm. The operational temperature of the chip was 213 K with a dark current less than 4 e^−^ pixel^−1^ min^−1^. The pixel layout was 1024 by 256, and the dynamic range of the 16 bit analog to digital converter was 65535 digital number (*DN*). The measurements were performed by binning the 256 pixels in the short direction of the CCD chip. The entrance slit of the monochromator was equipped with a mechanical shutter, the single axial Model 227MCD (JY Horiba). Appendix A describes the procedures used to validate the performance of the fluorometer [4].

### 3.1 Calibration of the Fluorometer With SRM 1932

SRM 1932 certifies the concentration of fluorescein as (60.97 ± 0.88) µmol/kg. Given the density of the SRM buffer as 1.003 g/mL, the molar concentration of the SRM 1932 is (61.15 ± 0.88) µmol/L. This value of the concentration was used as the initial concentration. Serial dilutions were made by combining previously made solution with additional buffer. All solutions were prepared gravimetrically using a calibrated balance (Sartorius 2024MP) with a resolution of 0.01 mg. The errors were obtained from the standard deviation of four weighins. The standard error in the fluorescein concentrations was about 1.6 % and originated mostly from the initial error in the SRM concentration. The weighin errors contributed a minimal error to the final fluorescein concentrations.

### 3.2 Measurement of the Fluorescence Signal

The fluorescein solution was pumped with a peristaltic pump through a capillary flow cell [5]. The 10× objective and the capillary were mounted on appropriate mounts to provide the necessary adjustment. A good adjustment was indicated by the appearance of a clean circular beam cross section after the transit through the capillary. Poor adjustment was characterized by complex interference bands from light reflected by various surfaces of the capillary. The capillary tube (length = 0.7 m) was coupled to a plastic tube (length = 20 cm) via a stainless steel pressure “Swagelok” coupling. The plastic tube was inserted into the pumping mechanism of a peristaltic pump (PP). A centrifuge vial (1.5 mL capacity) contained the test solution and a small magnetic stirring bar. The ends of the capillary or the plastic tube were inserted in the solution and the pump direction set appropriately to pump the solution to the waste container. Neither the capillary nor the plastic tube touched the waste solution. Formation and detachment of small droplets above the waste container was an indicator of flow.

The spectrum was accumulated over a period of 40 s leading to appearance of sharp spikes in the CCD response. The spikes are pixels with unusually large charge content, the spikes are confined to one or two adjacent pixels. We removed the spikes by simply replacing the contents of the pixel containing a spike (attributed to cosmic ray events hitting a pixel in the CCD array) by an average of the contents of two nearby pixels. The necessary overall dynamic range was obtained by varying the integration time from 0.5 s to 40 s, and by the dynamic range of the CCD itself (about 60). The ND filter was not used to modify the illumination intensity.

The peristaltic pump produced a pulsating flow with a time period of approximately 1.6 s. The strong illumination caused substantial photodegradation of the fluorescein solution in the capillary. The photodegradation and the pulsating flow produced a time variation in the fluorescence signal. This variation was averaged adequately during integration times longer then 5 s. However, for shorter integration times we had to take multiple measurements and average the resulting fluorescence signals. The pulsating fluorescence intensity enlarged the measurement errors for the concentrated fluorescein solutions. We avoided changing the intensity of the illuminating beam since that would change the photodegradation rate for measurements performed on solutions with different fluorescein concentrations. The assumption is made that the photodegradation is the same for solution and fluorescein immobilized on microspheres. To minimize possible systematic errors due to photodegradation, the flow conditions for the calibration and microsphere measurements were made as similar as possible. The polarization anisotropy for fluorescein solutions was approximately zero. Therefore, the measured spectra are characteristic of a solution of random emitters and systematic effects due to polarization are small.

### 3.3 Background Subtraction

**Fig. 2:**
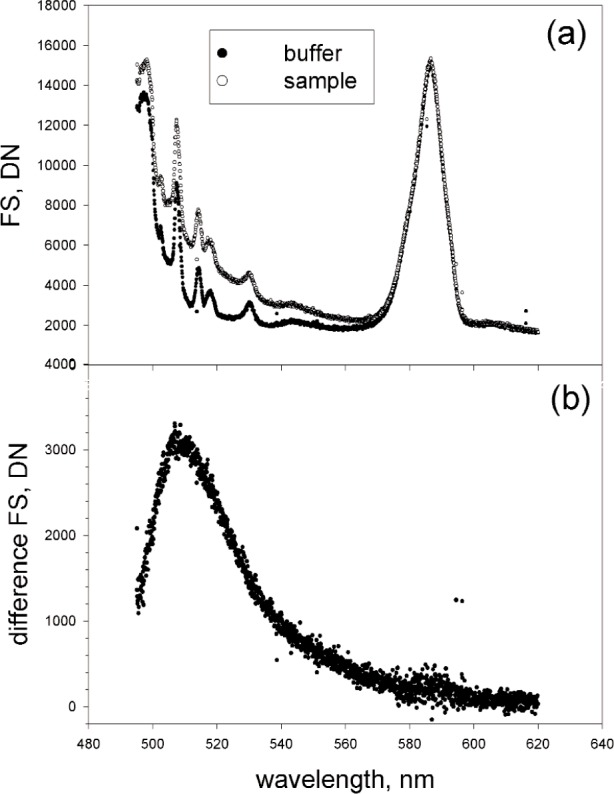
(a) The top trace is the recorded fluorescence spectrum from a solution with a fluorescein concentration of 16 pmol/L. The bottom trace is the spectrum from a pure buffer. The dominant features in both traces are the water Raman line at 585 nm and Raman lines from the capillary walls. Both traces were taken with a 40 s integration time. Spikes were eliminated by replacing the contents of a pixel with a spike by the average of the contents of several adjacent pixels. (b) The fluorescence from fluorescein determined by subtracting the bottom trace from the upper trace in (a). The quality of the subtraction is judged by the amount of residual Raman line. The integrated fluorescence signal (FS) was found by summing the subtracted trace. The sum is a good approximation of the integral over all wavelengths.

Figure 2a shows the measured spectra of a pure phosphate buffer (solid circles) and a fluorescein solution with a concentration of approximately 16 pM (open circles) in phosphate buffer. The laser line filter shown in Fig. 1 was a critical component in the fluorometer since without it the spectra in Fig. 2a would be an order of magnitude higher and dominated by laser plasma lines. The spectra in Fig. 2a have *DN* values larger than 2000, suggesting that CCD linearity correction was not significant. Figure 2b shows the spectrum when the buffer response is subtracted from the fluorescein solution response. As expected, the emission spectrum peaks at approximately 510 nm. The integration of the spectrum was performed by summing the *DN* values of the subtracted spectrum in Fig. 2b. The resulting truncation errors for wavelengths less than 595 nm and wavelengths greater than 620 nm were not evaluated. We estimate that these truncations lead to a systematic bias of less than −1 %. Similar spectra were collected for solutions with higher values of fluorescein concentration and the integration time was lowered as required to insure that the resulting CCD response was not saturated. The subtracted spectrum was corrected for the spectral response of the detector as described in Appendix A. The corrected integration times were used to normalize all integrated fluorescence signals (*FS*) to the fluorescence signal (*FS*) at an integration time of 1 s.

**Fig. 3:**
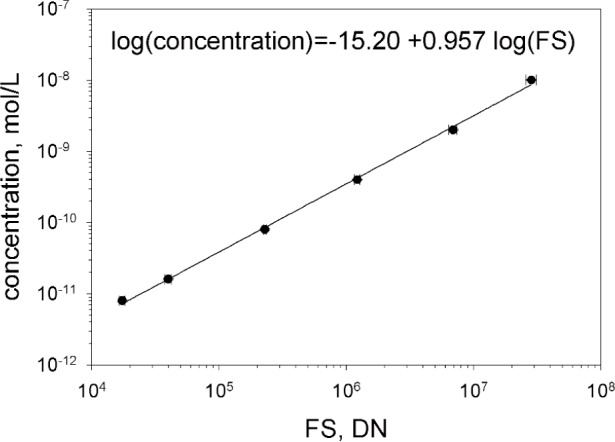
The plot of the log of the concentration of a fluorescein solution versus the log of the integrated fluorescence signal (*FS*) associated with the known concentration. The ideally linear response has a slope of 1.0. The linear relation between the logs of the two quantities constitutes a calibration of the fluorometer.

Figure 3 shows a log-log plot of the integrated *DN* values on the horizontal axis and five different concentrations of fluorescein plotted on the vertical axis. The best linear fit to the data in Fig. 3 is

log(concentration)=−15.20+0.957×log(FS) (11)

The errors of the fit parameters were 0.09 and 0.009 for the intercept and slope, respectively. The errors were obtained from a linear regression procedure in Mathcad. The lowest point (for a concentration of approximately 7 pmol/L) was systematically lower than expected from the linear trend set by the higher points. We rationalize this as an effect of adsorption of fluorescein on the capillary walls [4]. A calibration was accepted if the slope of the best fit fell between 0.95 and 1.05. A perfectly linear relation has a slope of 1.0, however a deviation of ± 0.05 was accepted. The values of “intercept” and “slope” are used in Eq. (10).

## 4. Measurement of Fluorescence From Microspheres With Immobilized FITC

The measurements of fluorescence signal were carried out in the identical apparatus as the calibration with serially diluted fluorescein solutions. Since the microsphere measurements were carried out after the fluorescein solution, great care was needed to eliminate possible contamination. Prior to the microsphere measurements, the capillary flow cell was washed for several hours. The washing was performed by pumping buffer through the capillary in alternating directions. The switch in pumping direction was important to clear possible dead spaces in the connection between the capillary tube and the plastic tube. As a rule of thumb, the cleaning was sufficiently good when the CCD signal was about 370 *DN* at 510 nm with an integration time of 10 s, and a power indicator of about 20. This number was obtained through experience. During all measurements the following pumping sequence was followed: first the suspension was pumped through the plastic tube into the capillary (this direction filled the capillary quickly); second the suspension was pumped through the capillary into the tube and fluorescence spectra were accumulated. Normal flow was indicated by the presence of a scattering diffraction pattern in the transmitted light. The pattern indicated the presence of spheres in the capillary as well as proper alignment. The intensity of the diffraction pattern fluctuated as expected since the number of microspheres in the sensing volume was of the order of twenty. On occasion, small bubbles passed through the illuminated region in the capillary. The passage of a bubble was obvious from the distortion of the transmitted laser beam. Clogging was obvious because flow stopped and the fluorescence signal decreased.

### 4.1 Fluorescence Measurement

**Fig. 4:**
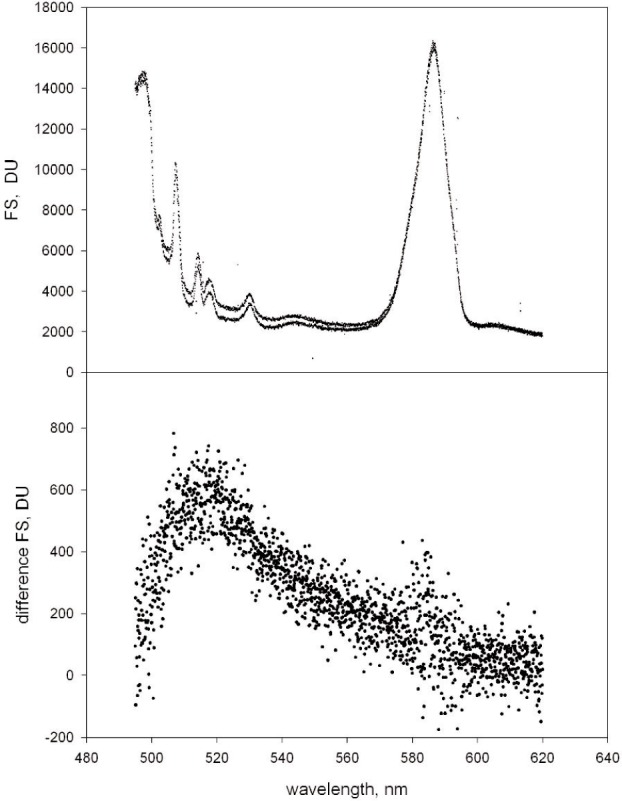
(a) The top trace is the recorded fluorescence spectrum from a suspension of microspheres with immobilized FITC. These are the microspheres with the smallest amount of FITC. The bottom trace is the spectrum from a suspension of microspheres with no FITC, “blank” microspheres. The dominant features in both traces are the water Raman line at 585 nm and Raman lines from the capillary walls. Both traces were taken with a 40 s integration time. Spikes were eliminated by replacing the contents of a pixel with a spike by the average of the contents of several adjacent pixels. (b) The fluorescence spectrum from microspheres determined by subtracting the bottom trace from the upper trace in (a). The integrated fluorescence signal (*FS*) was found by summing the subtracted trace.

Figure 4a shows the spectrum measured for a suspension of microspheres (Suspension #1, open circles) and a suspension of blank microspheres (solid circles). The suspensions were identical to those used in the microsphere concentration measurements. The measurements in Fig. 4a were performed with integrating time of 40 s, and power indicator displaying 19.2. Figure 4b shows the difference spectrum. The location of maximum emission shifts to the red, and the spectrum is broader. Both facts are typical of emission from immobilized FITC and serve as additional indicators that the flow cell is clean and free of fluorescein in solution. The quality of background subtraction was gauged by the disappearance of the water Raman line centered at 585 nm. In further analysis, the spectra in Fig. 4a were corrected for CCD non linearity (≈0.8 % effect) and normalized to an integration time of 1 s. The spectrum was summed and the errors of the summed spectrum were estimated from multiple measurements. Spectral response corrections were made on the data in Fig. 4 (≈3 % effect). The resultant summed spectrum for Suspension #1 was (11220 ± 600) *DN*. The value of the summed spectrum was substituted for FS in Eq. (10). Taking the ratio of average power levels during calibration and microsphere fluorescence measurements gave *P*_adj_ = 0.92 ± 0.02.

## 5. Measurement of Microsphere Concentration

The microsphere concentration was measured using a Coulter Multisizer 3 particle counter. The instrument detects small changes in conductivity between two reservoirs separated by a narrow orifice of diameter 100 µm. Whenever a particle passes through the orifice from one reservoir to the other, a transient change in conductivity is detected and recorded as a particle. In practice, the reservoir outside the orifice is the test reservoir into which samples are placed. A volumetric syringe withdraws fluid from the test reservoir through the orifice and the concentration is determined as the particle counts divided by the preset volume of fluid withdrawn. The fluid that was placed into the test reservoir was obtained from the original fluid by diluting the original fluid about 500 times using Isotone fluid. Isotone is a proprietary fluid which has optimal properties for the performance of the Multisizer 3. The composition of Isotone is sufficiently close to that of phosphate buffer saline (PBS) so that microsphere suspension stability is not degraded. The concentration of the particles in the original fluid is found by multiplying the measured concentration by the reciprocal of the dilution.

### 5.1 Determination of the Dilution Factor of the Test Suspension

The microspheres were obtained from Bangs Laboratories as a special order and were delivered in 5 mL opaque plastic bottles. The nominal solids mass fraction was about 2 %. The microspheres, as shipped, were suspended in a solvent optimized for enhancing the stability of the suspension. For the purpose of measuring the fluorescence signal of the microspheres, it was necessary to re-suspend the microspheres in pH = 7.2 PBS. The first step was to take about 1 ml of the microsphere suspension directly from the plastic bottle and centrifuge it (Marathon 13K centrifuge from Fisher Scientific at 2000 rpm). The pellet of microspheres was then re-suspended in 1 ml of PBS, pH 7.2, containing ≈0.1 mg SDS. An additional advantage of buffer exchange is that possible soluble fluorescent impurities in the bottle are minimized. The mass of about 200 µg of the resulting suspension was placed into a container with a mass of Isotone equivalent to about 100 ml of Isotone. The mass of the buffer was determined using a balance, OHaus ED4130, while the suspension was transferred using calibrated 100 µL or 200 µL pipettes and the mass approximated as 0.1996 g using the known density of water. A gravimetric check of the amount of solution delivered by the 100 µL pipette gave (0.10016 ± 0.0006) g using water at 23 °C and a Sartorius 2024 MP balance. The error in the reciprocal of the dilution factor was estimated to be less than 1 %. The remaining suspension was used for fluorescence measurements as described above.

### 5.2 Concentration Measurement

**Fig. 5:**
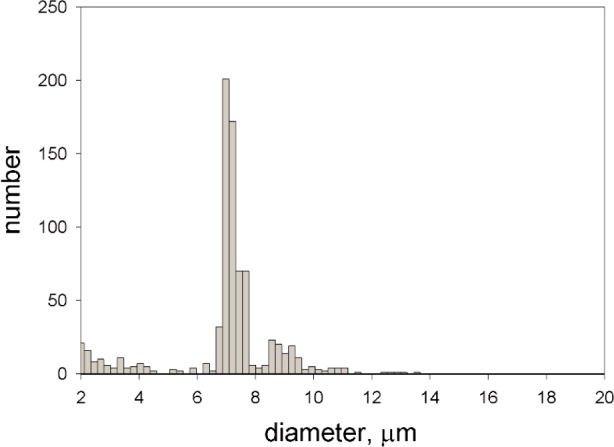
The frequency distribution of microspheres of specific size as determined by the Coulter Multisizer 3. The data was taken for a 500 µL sample of diluted suspension of the microspheres. Using the known dilution factor and the number of particles of the appropriate size, a concentration of microspheres was determined. The peak to the right of the major peak corresponds to microsphere “doublets” which are permanent associations of two microspheres. The concentration of “doublets” was also determined.

Prior to each series of measurements the operation of the Multisizer 3 was verified by running a suspension of calibration microspheres. The quality of the measurement was evaluated by measuring the mean diameter of the microspheres, and the coefficient of variation (CV) of the diameter values. The diluted microsphere suspension was placed in the Multisizer 3 and stirred at an indicated rate of 13 rpm. An image of the orifice was always examined to verify that the orifice was free from debris. The instrument analytical volume was set to 500 µL or 1 ml and a dilution factor (as determined above) was entered as a parameter in the acquisition software. Figure 5 shows a typical result of a single Multisizer 3 measurement. The horizontal axis gives the inferred particle diameter, and the vertical axis gives the number of times a particle with this diameter passed through the orifice. Using the dilution factor, the vertical axis can be converted into concentration. The inferred concentration of Suspension 1 was (1.427 ± 0.016) × 10^6^ mL^−1^ for all particles with diameters between 6.3 µm and 8.1 µm. The peak to the right of the main peak corresponds to “doublets”. These are events associated with the passage of two particles. The concentration of “doublets” (diameters between 8.3 µm and 9.7 µm) was calculated to be (0.077 ± 0.002) × 10^6^ mL^−1^. Since the concentration of particles in the test solution is small, it is likely that the “doublets” correspond to permanent associations of two microspheres. For permanent associations, the radii of doublets and triplets are related to the particle radius by *r*_doublet_ = 2^1/3^*r*_sphere_ and *r*_triplets_ = 3^1/3^*r*_sphere_ respectively. These relations are consistent with the observed size distributions of singlets, doublets, and triplets. This measurement of particle associations is relevant to the proper interpretation of the fluorescence signal as discussed below. The concentration measurement was repeated five times and the error associated with each concentration was found by dividing the standard deviation by the square root of 5, the number of trials. It should be noted that the error in the concentration is about 5 % implying that the error due to the uncertainty of the dilution factor (about 1 %) is negligible. The error of the concentration measurement is due mainly to the statistics of the particles entering the orifice. Whenever possible, the errors in the concentration measurement were minimized by using more concentrated test suspensions and larger sampling volumes.

### 5.3 Correction for Microsphere Doublets

Both the Multisizer 3 and the cytometer measurements show the presence of microsphere doublets- permanent associations of two microspheres. Some aggregation of colloidal particles is expected and the amount depends on previous treatments (e.g., sonication, age, and solvent). The concentration measurements were performed with the same suspension as the fluorescence measurements. Furthermore the two measurements were performed on the same day. The simplest assumptions are that the Multisizer 3 gives the correct concentration of doublets in the suspension used for fluorescence measurements and that the fluorescence intensity from doublets is twice the fluorescence intensity from single microspheres. Therefore, in comparing the fluorescence yield of a suspension and solution we multiply the doublet concentration by a factor of two relative to the singlet concentration.

$$N_{\text{Total}}=N_{\text{Single}}+2N_{\text{Double}}.$$ (12)

Where *N*_Single_ is the number concentration of single microspheres, *N*_Double_ is the number concentration of double microspheres, and *N*_Total_ is the total concentration which should be used in comparing fluorescence yields in Eq. (9). The assumption that the fluorescence intensity from a double microsphere is twice the fluorescence from a single microsphere is qualified by such consideration as distortion of the illuminating light and changes in quantum yield at point of contact between the microspheres. The measured polarization anisotropy in microsphere suspensions was about 0.08. This small value was rationalized by the large tether length of the chain of seven carbon atoms that immobilized the FITC to the microsphere surface. A long tether permits considerable rotational freedom. The systematic effects due to polarization differences between solution and microsphere suspension were neglected.

## 6. Assignment of *MESF* Values

The assignment of values of molecules of equivalent soluble fluorophore (*MESF*) entails the comparison of the fluorescence signal from suspensions of microspheres with immobilized fluorophore and solutions of fluorophore [3]. The comparison requires the integration of the emitted fluorescence over all wavelengths. The comparison requires a correction for differences in the molar absorption coefficient of soluble and immobilized fluorescein. Fluorescence excitation spectra show that the absorption spectra of the soluble and immobilized fluorescein are shifted relative to each other [3]. The fluorescence excitation spectra allow us to estimate the change in absorbance at 488 nm due to the shift. However, we were not able to measure the absolute value of the molar absorption coefficient for the immobilized fluorescein. This adjustment is left for a future refinement. The values of FS, “intercept”, “slope”, Padj, and *N*_spheres_ were used in Eq. (9) and Eq. (10) to calculate a value of *MESF* of 1667 ± 400. The same procedure was carried out for the other suspensions. Four independent series of measurements were carried out and the resulting *MESF* values averaged to give the final value reported in the Certificate of Investigation.

## 7. Linearization of *MESF* Assignments With a Cytometer

The objective of this measurement was to demonstrate that the *MESF* assignments obtained with the fluorometer were self-consistent. The microspheres were passed through a cytometer, and the response was measured. Each population of microspheres produced a population of fluorescence pulses which are characterized by a mean pulse area and a standard variation. Since the cytometer response is linear, the mean pulse height of the five populations of microspheres should correlate linearly with the assigned values of *MESF*. The cytometer does not provide a measurement of the absolute *MESF* values, however it does place a stringent constraint on relative *MESF* values. The *MESF* values obtained with the fluorometer were modified so that they correlate linearly with the mean channel measured with the cytometer. Appendix B gives details of the procedure used for validating the performance of the cytometer.

### 7.1 Microsphere Measurements

**Fig. 6:**
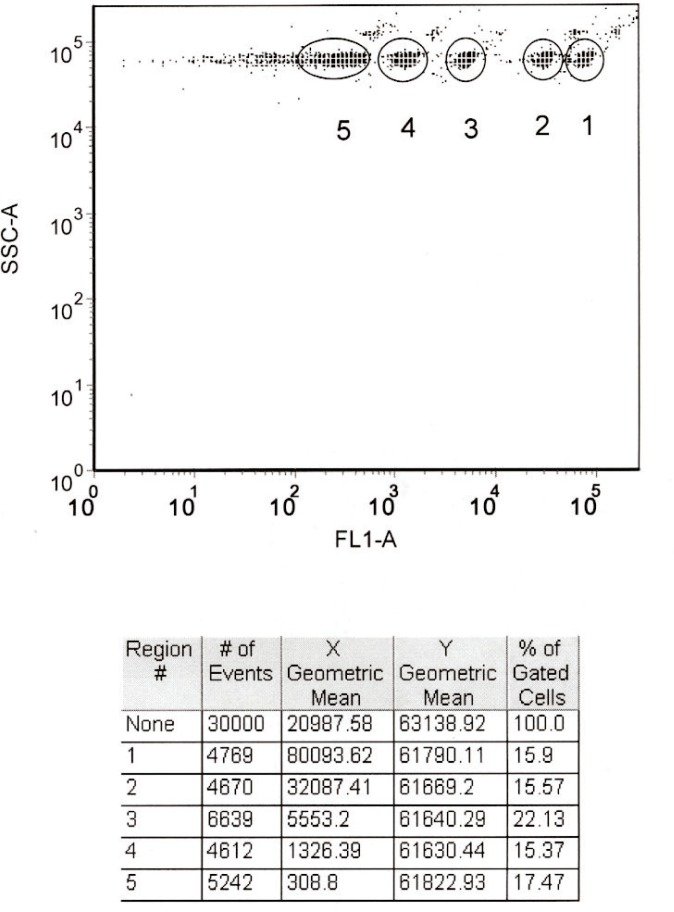
Output window from FCS Express V2, a program for the analysis of cytometer data. The upper diagram shows the distribution of side scattering signals (SSC) and fluorescence signals (FL1) from six populations of microspheres containing different amounts of immobilized FITC. The broad peak closest to the SSC-A axis is the signal from the blank microspheres with no immobilized FITC. The table below the diagram contains the geometric means of the scattering signal (*Y*) and fluorescence signal (*X*) for each of the five populations defined by the circular regions in the graph above. The row label “1” denotes the population with the highest fluorescence signal. The row labeled “None” contains the geometric means for the entire data set. Other parameters, such as the width of the distribution, can also be obtained for each population.

Alignment microspheres from Spherotech Corp. were used to align the cytometer laser beam. The alignment was sufficiently good when the fluorescence pulse mean channel was between 180 000 *DN* and 200 000 *DN*, and the CV was better than 4 %. After the alignment, the six populations of the microspheres in the reference material were mixed and pumped through the cytometer and the corresponding fluorescence and scattering peaks recorded. The suspensions were prepared by putting two drops of the suspension from each of the six plastic bottles into 1 ml of PBS buffer. Figure 6 shows typical results. Note that the single and double microsphere signals are resolved. The dense groupings with circular bounds correspond to single microsphere signals. Diagonally to the upper right of each dense grouping are less dense groupings (not enclosed by boundaries) corresponding to the passage of double microspheres. The table in Fig. 6 gives the properties of the groups of dots enclosed by the circular bounds. The *Y* geometric mean gives the mean scattering pulse amplitude, while the *X* geometric mean gives the mean fluorescence pulse height for each population. As expected, the scattering is relatively constant for the five populations, while the fluorescence signal differs substantially.

**Fig. 7:**
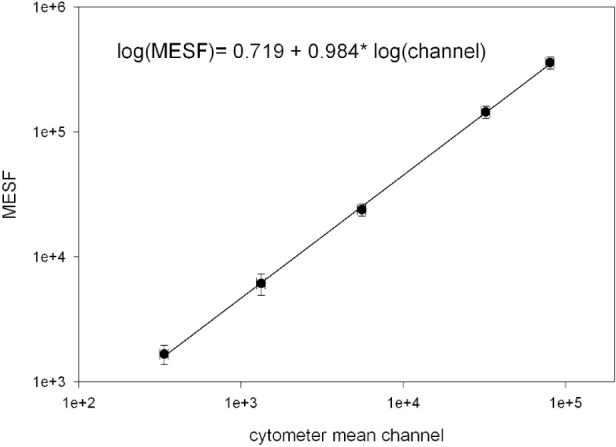
The plot of the log of the *MESF* value assigned to a given microsphere population versus the log of the mean channel determined by the cytometer (*X* in the Table in Fig. 6). The cytometer measurements provide a relative relation between the *MESF* values. The *MESF* values determined by the fluorometer were modified slightly to conform to the linear relation as given by the cytometer measurements.

Figure 7 shows a plot of the log of the mean fluorescence channel (*X* geometric means in Fig. 6) associated with each microsphere as a function of the log of the assigned *MESF* value for each microsphere. As described above, the *MESF* assignments were performed using the fluorometer. A fit to a straight line gives a slope of 0.984, indicating that there is a small deviations from linearity. All points are within two standard deviations of the best straight line fit. Since the cytometer is a linear device, the measured mean channels should correlate linearly with the *MESF* values which are proportionate to the number of fluorophore on the microsphere and hence to the fluorescence signal. This fact provides a means to linearize the assigned *MESF* values.

### 7.2 Linearization

The response of the cytometer was shown to be linear over the dynamic range encompassing the response of the five microsphere populations. Therefore, the *MESF* values have to correlate linearly with the observed mean channels in the cytometer measurement. The cytometer measurement provides a relative ordering of the *MESF* values but gives no measure of the absolute values. The procedure that was used to impose a linear relation on the *MESF* values determined by fluorometer was as follows. The solid circles in Fig. 7 show the *MESF* values obtained in Series 4 measurements as a function of mean cytometer channel. The data in Fig. 7 was fitted with a straight line whose slope was constrained to 1.027, which characterizes the cytometer linearity. Next, the *MESF* value given by the straight line was calculated for each mean channel. This calculated value is the linearized *MESF* value obtained for that series of measurements.

## 8. Certification of *MESF* Values

The *MESF* values were assigned by averaging the values obtained in four independent measurements each consisting of 1) calibration of the fluorometer using SRM 1932, 2) measurement of the microsphere fluorescence intensity, and 3) measurement of the microsphere concentration. The data in each set of four measurements gave an assignment of *MESF* values to the five populations of microspheres. The four different assignments provide a measure of reproducibility and an estimate of random error. The possible error obtained from the variation of the four *MESF* assignments was consistent with the error estimate for each of the four *MESF* assignments. The average values are reproduced in the Certificate of Investigation for RM 8640. The cytometer measurements were used to linearize the *MESF* values yielding values of linearized *MESF*. The four values of linearized *MESF* were averaged to give the average linearized *MESF* values in the Certificate of Investigation. The certificate includes both the average *MESF* values determined by the fluorometer, and the average linearized *MESF* values.

The *MESF* values were assigned under certain assumptions which are restated below in the order of decreasing relevance.

1. The molar extinction coefficient is the same for fluorescein in solution and fluorescein immobilized on the microspheres. It is known that there are differences in the wavelength of maximum absorption between fluorescein in solution and on the surface of the microsphere. It is expected that the magnitude at maximum absorption will also be different, however these have not been measured yet. The difference in molar extinction coefficient can be taken into account as soon as the values become available. The equality of fluorescence yield would imply that *N*_sus_ microspheres are equivalent to a concentration of soluble fluorophore given by *N*_sol_[*ε*_sol_(*λ*_x_)/*ε*_sus_(*λ*_x_)].
2. Photodegradation of fluorescein is the same in solution and at the surface of the microsphere. Systematic measurements of photodegradation in the two environments are not available. Differences in photodegradation rate would lead to systematic differences in the fluorescence signal between calibration measurements and microsphere measurements.
3. Residual polarization of fluorescence emission is the same for fluorescein in solution and at the surface of the microsphere. The measured polarization anisotropies are slightly different for fluorescein in solution and on the surface of a microsphere. Sensitivity of detectors to polarization would lead to small systematic differences in fluorescence signal between fluorescein in solution and on the surface of microspheres.
4. Adsorption on capillary flow cell walls is the same. If the adsorption (and holdup in dead spaces) of fluorescein in solution and fluorescein on microspheres is not the same, then there will be systematic differences in the fluorescence signal at low and high concentrations.
5. Illumination of FITC immobilized on microspheres is the same as the illumination of fluorescein in homogeneous solution.

## 9. Conclusions

A method was described for comparing the fluorescence yields of a solution of fluorescein and a suspension of microspheres with immobilized fluorescein isothiocyanate (FITC). The equality of fluorescence yields leads to an assignment of molecules of equivalent soluble fluorophore (*MESF*) to a microsphere with immobilized FITC. The *MESF* values may be the appropriate units for comparing fluorescence measurements. There is a need to investigate the validity of some of the assumptions that were made in carrying out the *MESF* assignment as described above.

## 10. Appendix A. Fluorometer Characterization

### 10.1 Wavelength Calibration

The wavelength calibration was performed using mercury lamp lines, and a neon lamp (Oriel 6032). The parameters in the data acquisition software were adjusted so that over the wavelength range 480 nm to 700 nm, the deviation between the true and measured wavelengths was less than 1 nm.

### 10.2 CCD Linearity

The output of a light emitting diode (LED) was passed through a variable ND filter and focused on the end of a bifurcated optical fiber. One of the arms of the optical fiber was placed at the location of the sample in the monochromator setup (see Fig. 1), and the other arm was placed in front of a photo diode (PD). Silicon photodiodes are known to be linear within 0.1 % up to a photocurrent of 200 µA [6]. The LED spectrum was recorded by the CCD for an integration time of 0.2 s. The spectrum was taken with automatic subtraction of the dark current. The CCD response was characterized by finding the average value of *DN* in a narrow range (518 nm to 522 nm) of wavelengths around the maximum response. The integrated CCD response was compared to the photo diode reading.

We examined the dependence of the average signal in *DN* as a function of the PD response. There was a substantial deviation from linearity at low values of *DN*. The most likely source of this deviation is trapping of electrons during the readout process [7]. During readout, the electrons are switched sequentially from pixel to pixel. The trapping centers prevent some of the electrons from reaching the final register pixel. Since the number of trapping sites is fixed, the relative importance of these sites increases with decreasing number of total electrons. The measured deviations provide a factor which can be used to linearize the CCD response. Thus the linearized *DN* value is given by

$$\begin{array}{l} {\left(DN\right)}_{\text{linear}}={\left(DN\right)}_{\text{measured}}(1+f)\\ \;f=10^{\left[0.6418-0.7181^{\prime }\log(DN_{\text{measured}})\right]} \end{array}$$ (1)

where (*DN*)_measured_ is the *DN* value that is read out during a measurement and (*DN*)_linear_ is the linearized value of the CCD response which is used in further data analysis. The factor *f* was obtained from the fit of the deviation of the measured CCD data from the linear PD response. We did not assign an error to the correction. The correction was applied to background measurements and sample measurements prior to subtraction of background. The correction is negligible for *DN* values above 600.

### 10.3 Integration Time Linearity

The CCD accumulates electrons for a preset integration time that is determined by a mechanical shutter located after the entrance slit of the monochromator. To measure the correspondence between the integration time setting in the software and the actual time, we illuminated the monochromator entrance slit with a constant light source and measured the CCD response for different indicated integration times *t*_indicated_. The reference light source (see Sec. 10.4) was used as the constant light source. The CCD response was integrated from 540 nm to 560 nm. The ratio of the CCD response divided by indicated time *t*_indicated_ relative to the CCD response for 1 s of indicated integration time showed a bias in the indicated time setting. At indicated times longer than 1 s, the actual integration time is shorter than the indicted time. The difference between the actual integration time, *t*_actual_, and the indicated integration time, *t*_indicated_, was corrected by multiplying the indicated time by the correction factor 1.00102–0.000782 × *t*_indicated_.

### 10.4 Spectral Response

The reference lamp’s output was calibrated at NIST over the range of wavelengths 340 nm to 800 nm in steps of 20 nm. The output port of the calibrated lamp was placed at the location of the sample in the fluorometer. The variable iris of the calibrated lamp was set so that the CCD response was between 10 000 *DN* and 40 000 *DN*. The CCD linearity is excellent in this region.

We formed the ratio of calibrated output of the reference lamp to the output at 520 nm (normalized reference output) and compared it to the ratio of measured CCD response of the reference lamp to the CCD response at 520 nm (normalized CCD response). The sharp decline in the response at shorter wavelengths was due to the holographic filter which was used to reject the 488 nm excitation light. The spectral correction factor was obtained by dividing the normalized reference output by the normalized CCD response to the reference lamp. Multiplying the measured CCD response by the correction factor corrects for the variability of the detector response over the wavelength range.

## 11. Appendix B. Cytometer Characterization

The cytometer was constructed to be as simple as possible with all physical processes open to inspection. An air-cooled Argon ion laser (Omnichrome Model 150) provided the source of 488 nm illumination. The laser beam was focused by a spherical lens with a focal length of 50 mm. The focal point was located in the flow channel of a cytometer flow cell provided by Becton Dickinson Biosciences. The sample was pumped by a syringe pump (Yale Apparatus Model YA-12), and the sheath fluid was pumped by the pressure in the container vessel. A flow meter (Aalberg Model TMR1-010426) in the sheath flow line gave an indication of the flow rate (usually set to 90 scale units). The laser beam passed through a glass plate whose orientation provided a sensitive adjustment of the beam position in the scattering plane. The flow cell contained the collection optics that focused the emitted light about 25 cm from the lens where photomultiplier (PMT) detectors (Hamamatsu Model H6780) were placed. The usual arrangement of dichroic mirror (DM) and bandpass filters selected the fluorescence and elastically scattered light components. The outputs from the two PMTs were processed by digital electronics provided by Becton Dickinson Biosciences (BD FACSDiVa system). An oscilloscope provided a visual monitor of the pulses associated with the side scattering (SSC) and the first fluorescence (FL1) channels of the detection electronics.

### 11.1 Linearity and Dynamic Range

The output of a green LED was focused on a slit of a chopper and then split by a glass plate reflector and passed to the inputs of two optical fiber (FO) bundles. One of the split beams (that transmitted through the glass plate) passed through a neutral density (ND) filter, and the FO routed the light to the PMT associated with the first fluorescence channel (FL1). The other beam (reflected from the glass plate) was incident on the FO that routed the light to the PMT associated with the side scattering channel (SSC). The chopper rotation was adjusted to give pulses that approximated the duration of the pulses from the microspheres in the flow cell. The adjustment was performed by visual inspection of the oscilloscope traces. The pulse rate was about 190 pulses per second. The linearity measurements were performed by noting the mean channel of the detected pulses in FL1 for a given OD value of the ND filter. Six ND filters were purchased from Newport Corp., and the attenuation values were used as provided by the manufacturer. The data was accumulated by recording events for different OD values of the ND filters. The six mean channels corresponding to OD values of 0, 0.51, 1.05, 1.50, 1.98. and 2.49 were recorded. The mean channels were plotted on a log-log scale versus the OD values. The average slope was found to be 1.027 ± 0.008. Therefore, we conclude that the cytometer response is linear. We assume that the photon pulses in the above simulation and from fluorescent microspheres behave in an identical fashion.

The noise properties of the cytometer are given by the coefficient of variation (CV) defined as the standard deviation of a pulse distribution divided by the mean pulse amplitude [8]. A linear relation between (CV)^2^ and the inverse of the mean pulse amplitude was observed, and indicated that the main source of noise was the statistics of photon arrival at the photomultiplier cathode.

## Footnotes

1 Certain commercial equipment, instruments, or materials are identified in this paper to foster understanding. Such identification does not imply recommendation or endorsement by NIST, nor does it imply that the materials or equipment identified are necessarily the best available for the purpose.

## References

[1] Gaigalas AK, Li L (2001). The Development of Fluorescence Intensity Standards. J Res Natl Inst Stand Technol.

[2] Prestwich WV (2003). Unit of Medical Physics.

[3] Schwartz A, Wang L (2002). Quantitating Fluorescence Intensity from Fluorophore: The Definition of *MESF* Assignment. J Res Natl Inst Stand Technol.

[4] Mavrodineanu R, Shultz JI (1973). Accuracy in Spectrophotometry and Luminescence Measurements.

[5] Li L-Q, Davis LM (1995). Rapid and efficient detection of single chromophore molecules in aqueous solution. Appl Opt.

[6] Kubarsepp T, Haapalinna A (1998). Nonlinearity measurements of silicon photodetectors. Appl Opt.

[7] Smith RM, Beletic JW, Amico P (1998). How Linear are Typical CCDs?. Experimental Astronomy.

[8] Chase ES, Hoffman RA (1998). Resolution of Dimly Fluorescent Particles: Measure of Fluorescence Sensitivity. Cytometry.

